# Combined *Lycium barbarum* Polysaccharides with Plasmon-Activated Water Affect IFN-γ/TNF-α Induced Inflammation in Caco-2 Cells

**DOI:** 10.3390/ph16101455

**Published:** 2023-10-13

**Authors:** Yu Zhi Lian, Yu-Chuan Liu, Chun-Chao Chang, Tomonori Nochi, Jane C.-J. Chao

**Affiliations:** 1 School of Nutrition and Health Sciences, Taipei Medical University, Taipei 110301, Taiwan; lian6239@hotmail.com; 2 Department of Biochemistry and Molecular Cell Biology, School of Medicine, Taipei Medical University, Taipei 110301, Taiwan; liuyc@tmu.edu.tw; 3 Cell Physiology and Molecular Image Research Center, Taipei Municipal Wan Fang Hospital, Taipei Medical University, Taipei 110301, Taiwan; 4 Division of Gastroenterology and Hepatology, Department of Internal Medicine, Taipei Medical University Hospital, Taipei 110301, Taiwan; chunchao@tmu.edu.tw; 5 Division of Gastroenterology and Hepatology, Department of Internal Medicine, School of Medicine, Taipei Medical University, Taipei 110301, Taiwan; 6 TMU Research Center for Digestive Medicine, Taipei Medical University, Taipei 110301, Taiwan; 7 Laboratory of Functional Morphology, Graduate School of Agricultural Science, Tohoku University, Sendai 980-8577, Japan; nochi@tohoku.ac.jp; 8 International Education and Research Center for Food and Agricultural Immunology, Graduate School of Agricultural Science, Tohoku University, Sendai 980-8577, Japan; 9 Master Program in Global Health and Health Security, Taipei Medical University, Taipei 110301, Taiwan; 10 Nutrition Research Center, Taipei Medical University Hospital, Taipei 110301, Taiwan

**Keywords:** ulcerative colitis, *Lycium barbarum* polysaccharides, plasmon-activated water, inflammation, apoptosis

## Abstract

The effects of *Lycium barbarum* polysaccharides (LBP) and plasmon-activated water (PAW) against IFN-γ/TNF-α induced inflammation in human colon Caco-2 cells were investigated. Cells were divided into the control, induction, LBP treatment (100–500 μg/mL), and combination groups with PAW. Inflammation was induced 24 h with 10 ng/mL IFN-γ when cell confluency reached >90%, and various doses of LBP with or without PAW were treated for 3 h, and subsequently 50 ng/mL TNF-α was added for another 24 h to provoke inflammation. Combination of LBP with PAW significantly decreased the secretion of IL-6 and IL-8. Cyclooxygenase-2 and inducible NO synthase expression was attenuated in all LBP-treated groups with or without PAW. NLRP3 inflammasome and related protein PYCARD expression were inhibited by LBP at the highest dose (500 μg/mL). All doses of LBP alone significantly decreased p-ERK expression, but combination with PAW increased p-ERK expression compared to those without PAW. Additionally, 250 and 500 μg/mL of LBP with or without PAW inhibited procaspase-3/caspase-3 expression. Therefore, LBP possesses anti-inflammation and anti-apoptosis by inhibiting the secretion of inflammatory cytokines and the expression of NLRP3 inflammasome-related protein. The combination with PAW exerts additive or synergistic effect on anti-inflammation.

## 1. Introduction

Gastrointestinal inflammation such as inflammatory bowel disease (IBD) was considered an autoimmune disease with the characteristics of prolonged exposure to inflammatory mediators and relapsing onset of disease severity [1], which could result in structure disruption, the impairment of intestinal functions, and abnormal immunological responses [2,3,4]. Pro-inflammatory cytokines such as tumor necrosis factor-α (TNF-α), interleukin (IL)-6, IL-8, and interferon (IFN)-γ and their mediators of inflammatory proteins such as cyclooxygenase-2 (COX-2) and inducible nitric oxide synthase (iNOS) were excessively expressed during the onset of disease, and these substances played central roles in promoting intestinal dysfunction [5,6]. NOD-, LRR- and pyrin domain-containing protein 3 (NLRP3) inflammasome could be activated by microbial infection, stress or toxic substances which could interact with apoptosis-associated speck-like protein containing a CARD (PYCARD), and PYCARD played crucial roles in host defense and inflammation, which was associated with the pathogenesis of IBD [7,8]. The protein expression of NLRP3 was increased by the activation of signaling pathway such as nuclear factor-κB (NF-κB) and extracellular signal-regulated kinase 1/2 (ERK1/2) which were involved in inflammation, differentiation, proliferation, survival, and apoptosis [9,10].

The treatment for IBD may vary depending upon the individuals. IBD patients were generally prescribed with multi-medication including anti-inflammatory drugs (such as mesalamine) and immuno-suppressors (such as cyclosporine), though the medication was given primarily to control disease severity. However, these medications may lead to several side effects including headaches, nausea, high blood pressure, kidney problems, and an increased risk of lymphoma [11,12]. Searching a safe and effective co-treatment for IBD from natural resources has recently become an alternative approach. *Lycium barbarum* berries, commonly known as goji berries, have been traditionally used in many nations as medicinal herbs and a food supplement, and possess various functional compounds such as polysaccharides, zeaxanthin, betaine, minerals, and vitamins [13,14]. *Lycium barbarum* polysaccharides (LBP) have demonstrated their biological therapeutic effects on antioxidation, anti-inflammation, immunomodulation, and neuroprotection [15]. Plasmon-activated water (PAW) was formed using resonantly illuminated gold nanoparticles (AuNPs) with deionized water by hot electron transfer, and possessed small water clusters and lower hydrogen bonding, which could enhance the solubility of lipophilic/hydrophobic substances, lower boiling point, and increase vapor pressure and osmosis [16]. Studies of PAW against diseases such as hepatic stress induced by chronic sleep deprivation and mice with non-small cell lung cancer were found to exhibit protective effects [17,18].

The effects of LBP against gastrointestinal disease including aspirin/ethanol-induced gastric ulcer and dextran sulfate sodium (DSS)-induced ulcerative colitis in rats were performed previously, and demonstrated positive outcomes against disease severity and promoted rehabilitation [19,20]. However, its underlying mechanisms remain elusive. Furthermore, the mixture of other substances such as C-phycocyanin and capsaicin did not exert additive or synergistic effects in our previous studies [19,20]; therefore, the change of solvent to PAW was further evaluated, and we assessed whether the mechanisms of LBP with PAW could act on anti-inflammation and/or anti-apoptosis using a cell model with the induction of inflammation. Hence, the objective of this study was to explore the possible mechanisms of LBP against IFN-γ/TNF-α-induced inflammation in human colon adenocarcinoma Caco-2 cells, and to further validate if the combination of LBP with PAW could show any additive or synergistic effects.

## 2. Results

### 2.1. LBP and Combination of PAW against IL-6 and IL-8 Secretion in Caco-2 Cells

**Figure 1:**
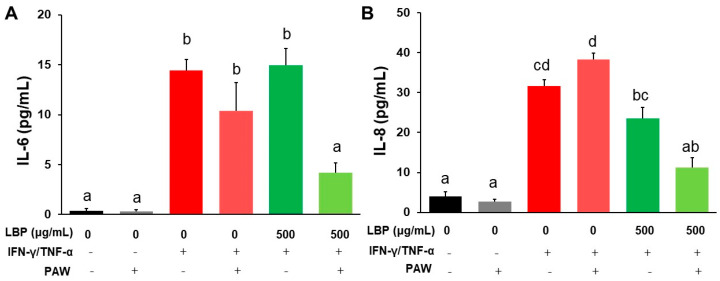
The levels of pro-inflammatory cytokines (**A**) IL-6 and (**B**) IL-8 in the medium of Caco-2 cells treated with *Lycium barbarum* polysaccharides (LBP, 500 μg/mL) with or without plasmon-activated water (PAW). Data are presented as mean ± SEM (*n* = 6 per group). The bars not sharing the same letter indicate statistical differences between the groups at *p* < 0.05.

After Caco-2 cells were provoked by IFN-γ/TNF-α, pro-inflammatory cytokines IL-6 (Figure 1A) and IL-8 (Figure 1B) in the medium with or without PAW were elevated compared to the PAW-control or control group, respectively (*p* < 0.05). A high dose of LBP (500 μg/mL) with PAW reduced IL-6 levels compared to the induction group with or without PAW and LBP at the same dose without PAW (Figure 1A). Cotreatment of LBP (500 μg/mL) with PAW decreased IL-8 levels compared to the induction group with or without PAW, but did not alter IL-8 levels compared to LBP at the same dose without PAW (Figure 1B). The treatment of LBP (250 μg/mL) with or without PAW decreased IL-6 levels compared to the induction group with or without PAW (*p* < 0.05) (Figure S1A), but 100 or 250 μg/mL LBP alone did not inhibit IL-8 levels compared to the induction group (Figure S1B). All doses of LBP combined with PAW showed significant decreases in IL-6 and IL-8 concentrations compared to the induction group with or without PAW (Figure 1 and Figure S1). LBP at a dose of 100 μg/mL with PAW decreased both IL-6 and IL-8 concentrations more effectively (Figure S1), indicating that the combination of PAW could exert additive or synergistic effect on decreasing pro-inflammatory cytokines.

### 2.2. Expression of Inflammatory Markers COX-2 and iNOS

**Figure 2:**
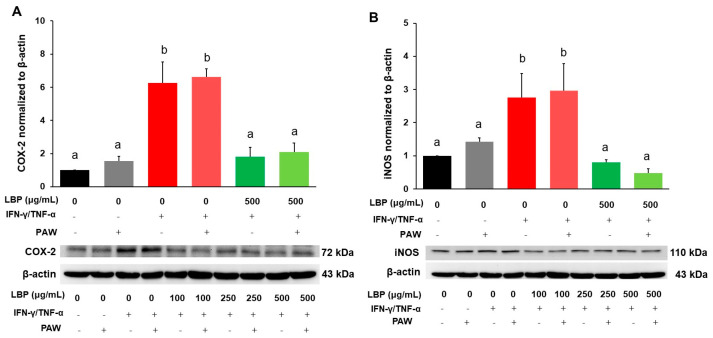
Regulation of *Lycium barbarum* polysaccharides (LBP, 500 μg/mL) with or without plasmon-activated water (PAW) against protein expression of inflammatory markers (**A**) COX-2 and (**B**) iNOS in Caco-2 cells. Data are presented as mean ± SEM (*n* = 5 per group). The bars not sharing the same letter indicate statistical differences between the groups at *p* < 0.05.

Protein expression of COX-2 (Figure 2A) and iNOS (Figure 2B) in Caco-2 cells was significantly elevated in the induction group with or without PAW compared to that in the corresponding control groups. The treatment of LBP at all doses with or without PAW showed significant decreases in COX-2 (Figure 2A and Figure S2A) and iNOS (Figure 2B and Figure S2B) protein expression compared to the corresponding induction groups, but there were no statistical differences among all LBP groups with or without PAW.

### 2.3. Expression of NLRP3 Inflammasome and PYCARD

**Figure 3:**
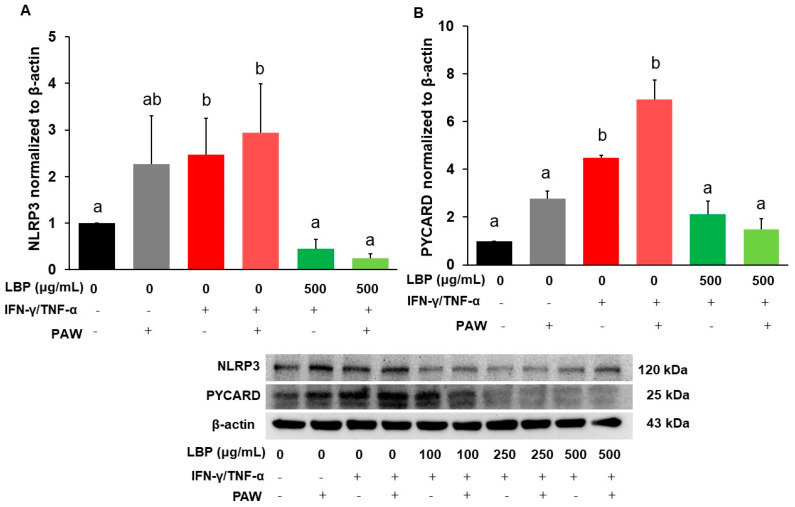
Modulation of *Lycium barbarum* polysaccharides (LBP, 500 μg/mL) with or without plasmon-activated water (PAW) against protein expression of (**A**) NLRP3 inflammasomes and (**B**) PYCARD in Caco-2 cells. Data are presented as mean ± SEM (*n* = 5 per group). The bars not sharing the same letter indicate statistical differences between the groups at *p* < 0.05.

Protein expression of NLRP3 inflammasome (Figure 3A) and PYCARD (Figure 3B) was significantly induced by IFN-γ/TNF-α compared to that in the control group (*p* < 0.05), but no statistical differences were shown between the induction group with or without PAW. The dosage of 250 μg/mL (Figure S3A) or 500 μg/mL (Figure 3A) LBP treatment alone inhibited protein expression of NLRP3 inflammasome, and LBP only at a dose of 500 μg/mL (Figure 3B) alone ameliorated protein expression of PYCARD compared to the induction group. The dosage of 250 μg/mL (Figure S3B) or 500 μg/mL (Figure 3B) LBP treatment with PAW suppressed protein expression of PYCARD compared to the induction group with PAW. However, LBP treatment with PAW did not exhibit any additive or synergistic effects on inhibiting protein expression of NLRP3 inflammasome and PYCARD compared to the corresponding LBP groups without PAW.

### 2.4. Expression of IκBα and ERK in Signaling Pathway

**Figure 4:**
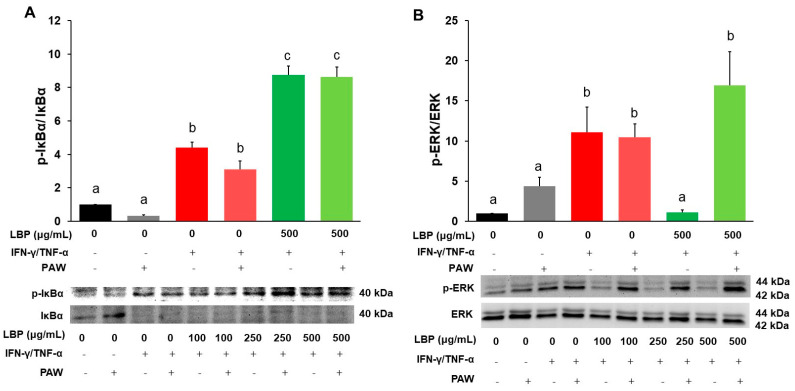
Regulation of *Lycium barbarum* polysaccharides (LBP, 500 μg/mL) with or without plasmon-activated water (PAW) against (**A**) the ratio of phosphorylated IκBα to total IκBα (p-IκBα/IκBα) and (**B**) the ratio of phosphorylated ERK to total ERK (p-ERK/ERK) protein expression in Caco-2 cells. Data are presented as mean ± SEM (*n* = 5 per group). The bars not sharing the same letter indicate statistical differences between the groups at *p* < 0.05.

Human colon Caco-2 cells induced with IFN-γ/TNF-α significantly increased both the protein ratio of phosphorylated IκBα to total IκBα (p-IκBα/IκBα) (Figure 4A) and phosphorylated ERK to total ERK (p-ERK/ERK) (Figure 4B) in the induction group with or without PAW compared to the corresponding control group, but there were no statistical differences between the induction groups with and without PAW. Treatment of LBP at lower doses alone did not exhibit any effects on p-IκBα/IκBα compared to the induction group (Figure S4A), but a high dose of LBP (500 μg/mL) with or without PAW elevated p-IκBα/IκBα compared to the corresponding induction groups (*p* < 0.05) (Figure 4A). All LBP treatment doses without PAW suppressed p-ERK/ERK compared to the induction group with or without PAW (Figure 4B and Figure S4B). However, LBP at all doses with PAW increased p-ERK/ERK compared to the control group with or without PAW and the corresponding LBP groups without PAW.

### 2.5. Expression of Apoptotic Related Markers

**Figure 5:**
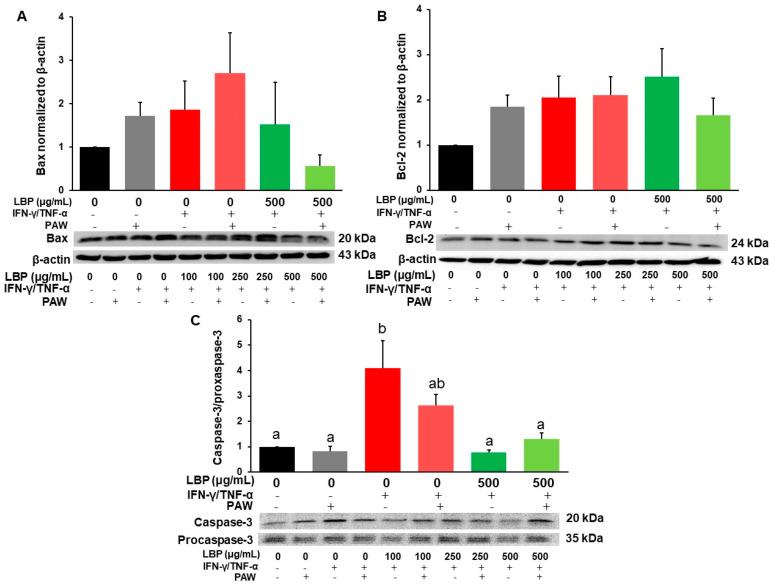
Modulation of *Lycium barbarum* polysaccharides (LBP, 500 μg/mL) with or without plasmon-activated water (PAW) against protein expression of apoptotic markers (**A**) Bax, (**B**) Bcl-2, and (**C**) caspase-3/procaspase-3 in Caco-2 cells. Data are presented as mean ± SEM (*n* = 5 per group). Protein expression of Bax and Bcl-2 was not significantly different among the groups (*p* > 0.05). The bars not sharing the same letter indicate statistical differences between the groups at *p* < 0.05.

Expression of apoptotic-related proteins Bax (Figure 5A and Figure S5A) and Bcl-2 (Figure 5B and Figure S5B) was not significantly different among all the groups. Protein ratio of caspase-3/procaspase-3 was significantly elevated in the induction groups with and without PAW compared to the control group without PAW (Figure 5C). The induction group with PAW tended to increase but not significantly change caspase-3/procaspase-3 compared to the control group with PAW. Higher doses of LBP (250 μg/mL in Figure S5C or 500 μg/mL in Figure 5C) with or without PAW significantly inhibited caspase-3/procaspase-3 compared to the induction group without PAW, but LBP combined with PAW did not exert significant effects compared to the corresponding LBP groups.

## 3. Discussion

The imbalance between pro- and anti-inflammatory cytokine profile is found in IBD, which could trigger oxidative stress, pain signaling, the loss of gut-barrier functions, and the disruption of intestinal epithelium balance [21,22,23]. High levels of TNF-α secreted by the lamina propria of the innate immune cells were found in IBD patients [24], which could stimulate TNF-α receptors and further activate NF-κB and ERK1/2 signaling pathways to initiate or propagate inflammation [25,26]. As stated in previously, the positive outcomes of LBP towards gastrointestinal diseases in rats was performed, but none of the studies presented any additive/synergistic effects when combined with other compounds [19,20], hence the possibility of changing the use of solvent was evaluated in our cell study. IFN-γ/TNF-α were used to induce cell inflammation and stimulate the secretion of pro-inflammatory cytokines IL-6 and IL-8 for mimicking inflammation in human colon cells. In our study, the pretreatment with 250 μg/mL LBP successfully decreased IL-6 levels in the medium. Similarly, Li et al., demonstrated that hot-water-extracted LBP at a dose of 200 or 400 μg/mL inhibited IL-6 and IL-8 release by inflamed Caco-2 cells with 100 ng/mL TNF-α induction [27]. The result was also shown that LBP (100 μg/mL) combined with PAW further lowered IL-6 and IL-8 levels, demonstrating that the combination of LBP with PAW may potentially have an additive or synergistic action on suppressing the secretion of pro-inflammatory cytokines in the inflamed colon cells.

Levels of prostaglandin E2 (PGE_2_) and nitric oxide (NO) were overproduced in the intestinal lumen and biological fluids during an active stage of IBD, which was in response to the activation of COX-2 and iNOS as the inflammatory markers for various inflammatory diseases [28,29]. Therefore, the attenuation of excessive PGE_2_ and NO by inhibiting the activation of COX-2 and iNOS is crucial for the therapy of IBD [28,29]. Our present study demonstrated that inflamed Caco-2 cells pretreated with LBP inhibited COX-2 and iNOS proteins, but such effects were not further enhanced by the combination of PAW because protein expression was decreased to the levels similar to those in the control group. Similarly, water-extracted *Lycium barbarum* at a dose of 500 or 1000 μg/mL suppressed mRNA and protein expression of COX-2 and iNOS in lipopolysaccharide (LPS)-induced RAW 264.7 cells [30]. These results indicate the anti-inflammatory effects of LBP.

NLRP3 inflammasome played a critical role in host defense as the first line of the innate immune system against microbial infection, and recent studies have shown the correlation between NLRP3 inflammasome and the development of IBD [31,32]. Irregular expression of NLRP3 inflammasome could promote the activation of pro-inflammatory cytokines such as IL-1β and IL-18, which might be involved in the pathogenesis of IBD [7], and the activation of NF-κB and ERK1/2 signaling pathways could also enhance the protein expression of NLRP3 inflammasome [33,34]. A previous study found that the inhibition of NLRP3 inflammasome was accompanied by decreases in the secretion of IL-1β, IL-18, and IL-33 in inflamed Caco-2 cells induced by TNF-α [35]. Our present study demonstrated that LBP at a dose of 500 μg/mL inhibited the protein expression of NLRP inflammasome and PYCARD in IFN-γ/TNF-α induced Caco-2 cells.

Interestingly, the pretreatment with LBP did not inhibit p-IκBα protein expression, but LBP at a high dose (500 μg/mL) could further provoke the activation of NF-κB, suggesting that the anti-inflammatory mechanism of LBP against IFN-γ/TNF-α induction in Caco-2 cells could not be correlated with the suppression of NF-κB activation. The treatment of LBP alone was found to inhibit the protein expression of p-ERK in IFN-γ/TNF-α induced Caco-2 cells, but the combination of PAW increased protein expression of p-ERK. A previous study demonstrated that water-extracted *Lycium barbarum* at a dose of 100–1000 μg/mL dose-dependently decreased the protein expression of p-IκBα and p-ERK in LPS-induced RAW 264.7 cells, indicating that the anti-inflammatory effects of water-extracted *Lycium barbarum* could act through the suppression of NF-κB and ERK1/2 signaling pathways [30].

ERK was involved in various phosphorylated targets which are responsible for cell survival and proliferation [36,37]. There is still a contradiction regarding the treatment of LBP against the activation of ERK. A previous study showed that the treatment of LBP increased p-ERK expression to avoid apoptosis in PC-12 neuronal cells with L-glutamate-induced toxicity [38]. However, our previous study found that LBP was capable of decreasing phosphorylation of ERK and had anti-apoptotic effects in RGM-1 gastric cells with aspirin-induced lesions [39]. Thus, apoptotic markers such as Bax, Bcl-2, and caspase-3/procaspase-3 expression were further evaluated. The over-reaction of apoptosis was found in the colonic lamina propria of ulcerative colitis patients, which could be triggered by oxidative stress and the inflammatory signaling pathway [40,41]. Previous animal studies also demonstrated that the dysregulation of apoptosis was found in mice with DSS-induced ulcerative colitis, which may cause the dysfunction and impairment of the intestinal barrier function [42,43]. Though the result was found that LBP had no modulatory effects on the protein expression of Bax and Bcl-2, but was able to inhibit caspase-3/procaspase-3 expression in the LBP groups treated at higher doses with or without PAW. Therefore, the treatment of LBP with or without PAW demonstrated potential anti-apoptotic effects via the inhibitory activation of caspase-3/procaspase-3 expression in inflamed Caco-2 cells.

The powder of LBP used in this study was commercially available with a concentration of approximately 50%, and such a concentration was similarly found in other studies [36,37]. The pretreatment of LBP powder (>50% purity) at a dose of 300 μg/mL successfully reversed oxidative stress and suppressed mRNA and protein expression of pro-inflammatory cytokines via the inhibition of the mitogen-activated protein kinase pathway in *E. coli*-infected primary bovine mammary epithelial cells [44]. The treatment of LBP (100 mg/kg bw) with a concentration of 43.65% for 7 days improved colitis symptoms, decreased inflammatory cytokine secretion in the plasma, and increased the gut abundance of *Akkermansia* and *Bifidobacterium* in mice with dextran sulfate sodium-induced colitis [45]. The purity of commercially available LBP powder ranged from 20% to 60%, and exerted positive results in various disease models [46,47,48,49,50]. The composition of LBP was not analyzed because maltodextrin was added as an excipient; hence, the purification procedure may be challenged because the hydrolysis of maltodextrin could potentially alter the composition of LBP.

The property and activity of liquid water predominantly depend on hydrogen bonding strength with the characteristics of donor–bridge–acceptor for proton transfer and electron donation [51,52]. The process of PAW altered its bonding strength to decrease polarity. The characteristics of PAW with better solubility, lower boiling point, higher vapor pressure, and higher osmosis [16] could potentially extract more nutrients or bioactive substances from raw materials [53]. The characteristics of PAW such as electron-doping and reduced hydrogen bonding strength could be maintained after aging for 3 days [53], and PAW was recommended to be used within 1 week. The innovation of PAW was considered novel, but its application against diseases or benefits to the regulation of physiological functions remains unknown. A previous study demonstrated that the consumption of PAW replacing drinking water had anti-inflammatory effects and could be beneficial to the relative abundance of specific probiotics such as *Akkermansia muciniphila* and other butyrate-producing bacteria in mice with colitis induced by 2,4,6-trinitrobenzenesulfonic acid [54]. Additionally, the combination of cisplatin and PAW in drinking water extended survival time in lung cancer mice-implanted LCC-1 cells compared to the combination of cisplatin and deionized water, and the possible mechanisms could be associated with the anti-inflammatory and antioxidant properties of PAW, which was proven by inducing nuclear factor erythroid 2-related factor 2 (Nrf2) antioxidant gene in human gingival fibroblasts [18].

This is the first study to investigate the application of PAW alone or with natural substances such as LBP in inflamed colon cells. The results demonstrated that PAW per se did not have any positive effects against IFN-γ/TNF-α induced inflammation in Caco-2 cells, but the combination with LBP showed better inhibitory effects on the secretion of pro-inflammatory cytokines. The results for the effects of LBP alone or LBP and PAW on protein expression of p-ERK in inflamed Caco-2 cells were not consistent, and further studies are required to verify the possible mechanisms considering LBP compositions, the dosage and incubation duration of LBP, and the bioavailability of LBP in the presence of PAW.

## 4. Materials and Methods

### 4.1. Materials and Reagents

IFN-γ (#300-02) and TNF-α (#300-01A) were bought from PeproTech Inc. (Rocky Hill, NJ, USA). Commercial LBP powder (M-5000) with a final concentration of 50% LBP was prepared by water extraction, and purchased from Fengyang Biomedical Co., Ltd., (Taichung, Taiwan). Plasmon-activated water was generously provided by Prof. Yu-Chuan Liu, and the preparation of PAW was described elsewhere [17]. Briefly, deionized water passed through a tube which was coated with gold nanoparticles (10 nm)-adsorbed ceramic particles under resonant illumination, resonant illumination was performed by green light-emitting diodes with a wavelength maximum centered at 530 nm to produce hot electron transfer and further break the hydrogen bonds of deionized water, and PAW was collected in glass bottles and used within one week. Cytokine IL-6 (DY206) was purchased from R&D systems, Inc. (Minneapolis, MN, USA), and IL-8 (431504) was bought from BioLegend (San Diego, CA, USA). Primary antibody iNOS (18985-1AP) from Proteintech Group, Inc. (Rosemont, IL, USA), COX-2 (ab15191) from Abcam (Cambridge, UK), NLRP3 (DF7438), PYCARD (DF6304), phosphorylated nuclear factor of kappa light polypeptide gene enhancer in B-cells inhibitor p-IκBα (AF2002), and total IκBα (AF5002) from Affinity Biosciences (Cincinnati, OH, USA), p-ERK (sc-81492) and total ERK (sc-514302) from Santa Cruz Biotechnology, Inc. (Dallas, TX, USA), Bcl-2-associated X (Bax) (CST #2772S) from Cell Signaling Technology (Danvers, MA, USA), and B-cell lymphoma 2 (Bcl-2) (IR94-392) from iReal Biotechnology, Inc. (Hsinchu, Taiwan) were used in this study.

### 4.2. Inflammation Induction and Treatments in Caco-2 Cells

**Figure 6:**
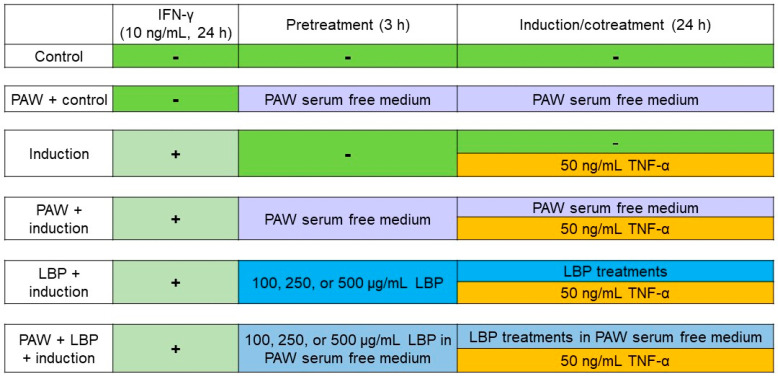
Overview design for *Lycium barbarum* polysaccharides (LBP) treatments, induction with interferon-γ (IFN-γ)/tumor necrosis factor-α (TNF-α), and use of plasmon-activated water (PAW)-prepared medium.

Human colon adenocarcinoma Caco-2 cells were maintained in Dulbecco’s modified Eagle’s medium supplemented with 10% fetal bovine serum, 1% non-essential amino acids, 6 mM glutamine, and 1% antibiotic antimycotic solution in a collagen-coated flask. Cells were kept in the incubator containing 5% CO_2_ at 37 °C, the medium was changed every 2–3 days, and cells were further sub-cultured when the confluency reached 70–80%. Cells were treated as follows: control, IFN-γ/TNF-α induction, IFN-γ/TNF-α induction and pretreatment with 3 doses of LBP (LBP + induction), and the corresponding 5 groups mentioned above with PAW replacing double-distilled water in serum-free medium. Cells (2 × 10^5^) were seeded into a 6-well plate till reaching 90% confluency, introduced with 10 ng/mL IFN-γ in serum-free medium with or without PAW to upregulate TNF receptor 2 expression, treated with 100, 250, or 500 μg/mL LBP in serum-free medium with or without PAW for 3 h, and then continuously incubated with the treatments and 50 ng/mL TNF-α for 24 h to induce inflammation [55]. The dosage of LBP was referred to a previous study using 3-(4,5-dimethylthiazol-2-yl)-5-(3-carboxymethoxyphenyl)-2-(4-sulfophenyl)-2H-tetrazolium (MTS) assay in C2BBe1 cells (a clone of Caco-2) [56]. Medium and cell pellets were collected for analysis, and the details for the treatments were shown in Figure 6.

### 4.3. Measurements of IL-6 and IL-8

Pro-inflammatory cytokines IL-6 and IL-8 were determined using enzyme-linked immunosorbent assay. Briefly, capture antibody was coated in a 96-well plate for 16–18 h, after several washes and coating, and medium was added for 1–2 h. Biotin conjugated secondary antibody, horseradish peroxidase conjugated with avidin, and substrates for the reaction were added subsequently according to the instruction of the manufacture, and the absorbance was detected at 450 nm after adding stop solution. 

### 4.4. Western Blot for Inflammatory and Apoptotic Proteins

Inflammatory proteins or mediators such as COX-2, iNOS, NLRP3 inflammasome, PYCARD, IκBα, and ERK and apoptotic proteins such as Bax, Bcl-2, procaspase-3, and caspase-3 were assessed by Western blot. Proteins in cell pellets were extracted using radioimmunoprecipitation assay buffer containing 1× protease inhibitor and 1× phosphatase inhibitor, further homogenized by ultrasonication, and centrifuged to collect the supernatant. Protein concentrations were determined by Lowry’s method [57]. Proteins (25–30 μg) were isolated by 10% sodium dodecyl sulfate-polyacrylamide gel electrophoresis, and blotted to methanol activated polyvinylidene fluoride membrane. The blots were blocked with 3% bovine serum albumin, subsequently probed with corresponding primary antibody at 4 °C overnight, and further incubated with specific secondary antibody for 1.5 h at room temperature. Protein bands were determined by enhanced chemiluminescence solution, and visualized by the imaging system (ChemiDoc-It 515 Imaging System Vision Works 8.18, UVP, LLC., Upland, CA, USA). Each protein band was quantitated by Image Pro Plus 4.5 software (Media Cybernetics, Inc., Bethesda, MD, USA).

### 4.5. Statistical Analysis

Data are indicated as mean ± SEM, and statistical data were analyzed by SPSS 19.0 (IBM Corp., Armonk, NY, USA). Statistical comparisons were assessed by one-way analysis of variance (ANOVA) and Tukey’s test. Statistical difference was considered at the level of *p* < 0.05.

## 5. Conclusions

The treatment of LBP exhibits anti-inflammatory effects by inhibiting IL-6 and IL-8 secretion and the suppressing protein expression of COX-2, iNOS, NLRP3 inflammasome, and PYCARD, as well as anti-apoptotic action by attenuating caspase-3/procaspase-3 expression in inflamed Caco-2 cells. Furthermore, the combination of LBP with PAW has a potential additive or synergistic effect on further decreasing IL-6 and IL-8 secretion. Therefore, the application of LBP against IBD symptoms seems to be promising, and the combination of PAW could be an additive or synergistic candidate for anti-inflammation.

## Data Availability

Data will be available upon request.

## Supplementary Materials

The following supporting information can be downloaded at: https://www.mdpi.com/article/10.3390/ph16101455/s1, Figure S1: The levels of pro-inflammatory cytokines (A) IL-6 and (B) IL-8 in the medium of Caco-2 cells treated with *Lycium barbarum* polysaccharides (LBP, 100–250 μg/mL) with or without plasmon-activated water (PAW); Figure S2: Effects of *Lycium barbarum* polysaccharides (LBP, 100–250 μg/mL) with or without plasmon-activated water (PAW) on protein expression of inflammatory markers (A) COX-2 and (B) iNOS in Caco-2 cells; Figure S3: Effects of *Lycium barbarum* polysaccharides (LBP, 100–250 μg/mL) with or without plasmon-activated water (PAW) on protein expression of (A) NLRP3 inflammasomes and (B) PYCARD in Caco-2 cells; Figure S4: Effects of *Lycium barbarum* polysaccharides (LBP, 100–250 μg/mL) with or without plasmon-activated water (PAW) on (A) the ratio of phosphorylated IκBα to total IκBα (p-IκBα/IκBα) and (B) the ratio of phosphorylated ERK to total ERK (p-ERK/ERK) protein expression in Caco-2 cells; Figure S5: Effects of *Lycium barbarum* polysaccharides (LBP, 100–250 μg/mL) with or without plasmon-activated water (PAW) on protein expression of apoptotic markers (A) Bax, (B) Bcl-2, and (C) caspase-3/procaspase-3 in Caco-2 cells.

## Abbreviations

ANOVA	analysis of variance
AuNPs	gold nanoparticles
Bax	Bcl-2-associated X
Bcl-2	B-cell lymphoma 2
COX-2	cyclooxygenase-2
DSS	dextran sulfate sodium
ERK1/2	extracellular signal-regulated kinase 1/2
IBD	inflammatory bowel diseases
IFN-γ	interferon-γ
IκBα	nuclear factor of kappa light polypeptide gene enhancer in B-cells inhibitor
IL	interleukin
iNOS	inducible nitric oxide synthase
LBP	*Lycium barbarum* polysaccharides
MTS	3-(4,5-dimethylthiazol-2-yl)-5-(3-carboxymethoxyphenyl)-2-(4-sulfophenyl)-2H-tetrazolium
NF-κB	nuclear factor-κB
NLRP3	NOD-, LRR- and pyrin domain-containing protein 3
PAW	plasmon-activated water
PYCARD	apoptosis-associated speck-like protein containing a CARD
SEM	standard error of mean
TNF-α	tumor necrosis factor-α
