# Supplementary material for: Combined Lycium barbarum Polysaccharides with Plasmon-Activated Water Affect IFN-γ/TNF-α Induced Inflammation in Caco-2 Cells
Source: Pharmaceuticals (Basel). 2023 Oct 13;16(10):1455. doi: 10.3390/ph16101455 (PMC10610401; doi:10.3390/ph16101455)
Supplement: Supplementary file 1 [file pharmaceuticals-16-01455-s001.zip › pharmaceuticals-2626155-supplementary.pdf]

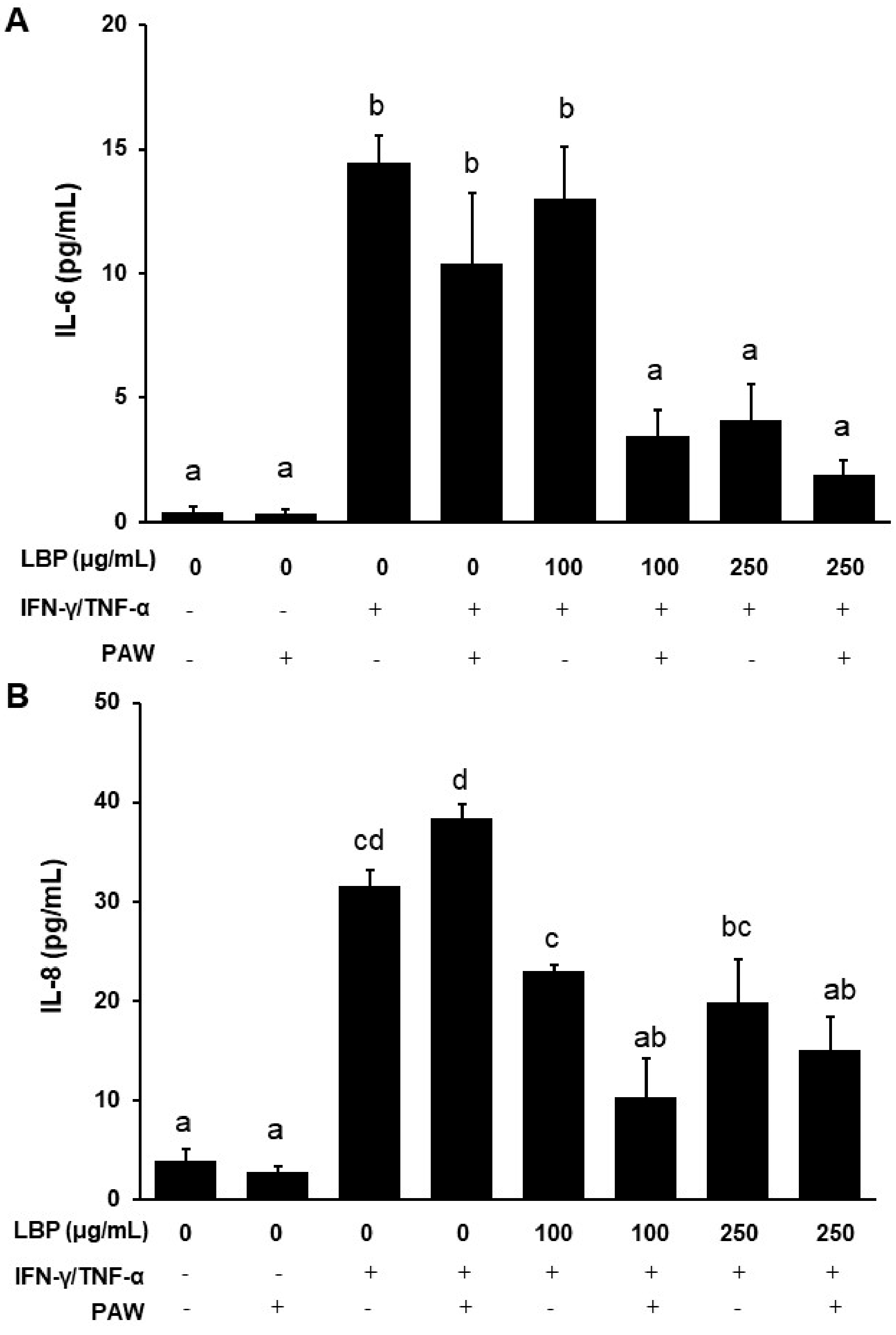

Figure S1. The levels of pro-inflammatory cytokines (**A**) IL-6 and (**B**) IL-8 in the medium of Caco-2 cells treated with *Lycium barbarum* polysaccharides (LBP, 100-250 µg/mL) with or without plasmon-activated water (PAW). Values are expressed as mean ± SEM (n = 6 per group). The data not sharing the same letter represent significant differences between the groups at $p < 0.05$.

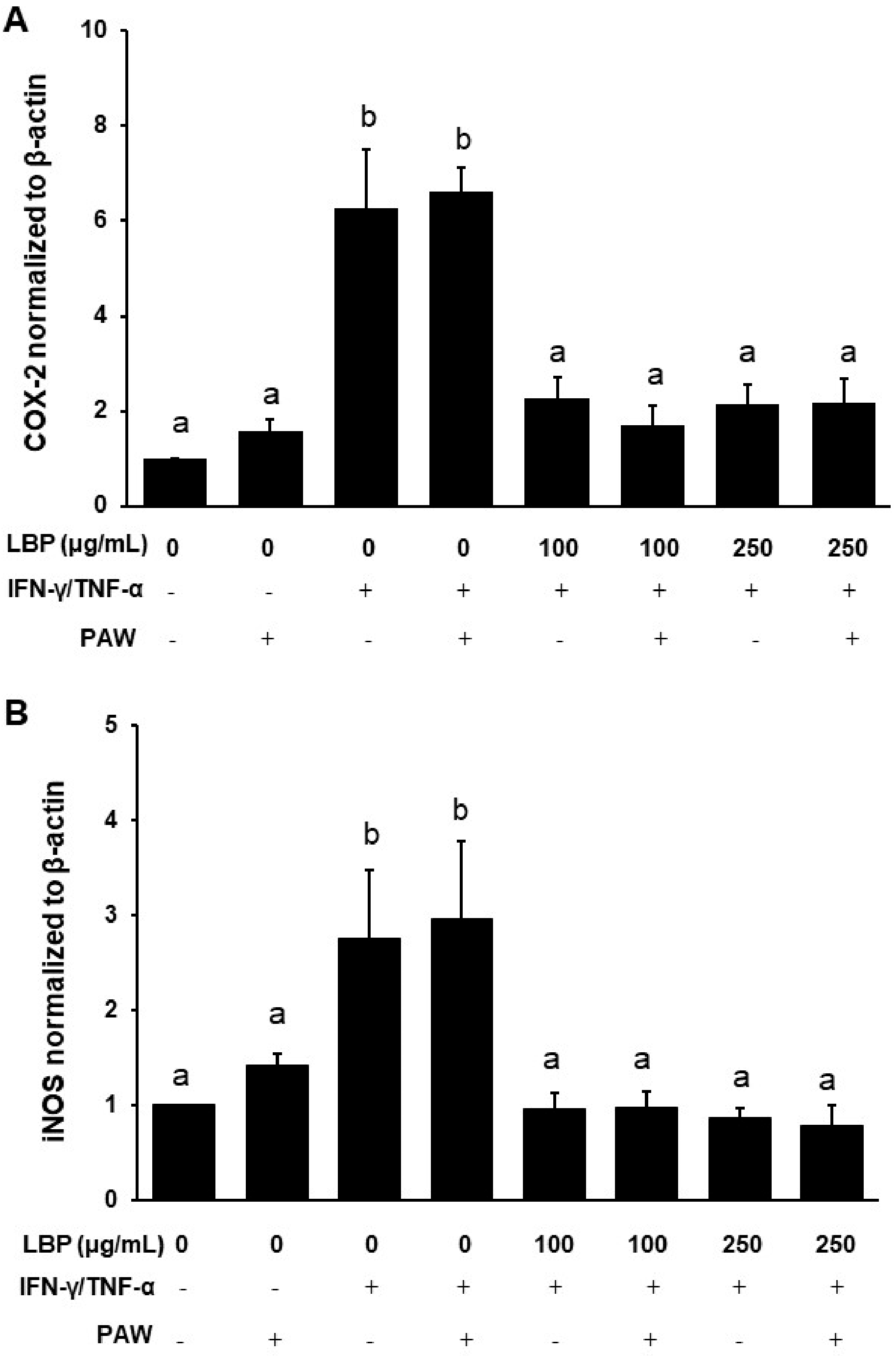

Figure S2. Effects of *Lycium barbarum* polysaccharides (LBP, 100-250 μg/mL) with or without plasmon-activated water (PAW) on protein expression of inflammatory markers (**A**) COX-2 and (**B**) iNOS in Caco-2 cells. Values are expressed as mean ± SEM (n = 5 per group). The data not sharing the same letter represent significant differences between the groups at $p < 0.05$.

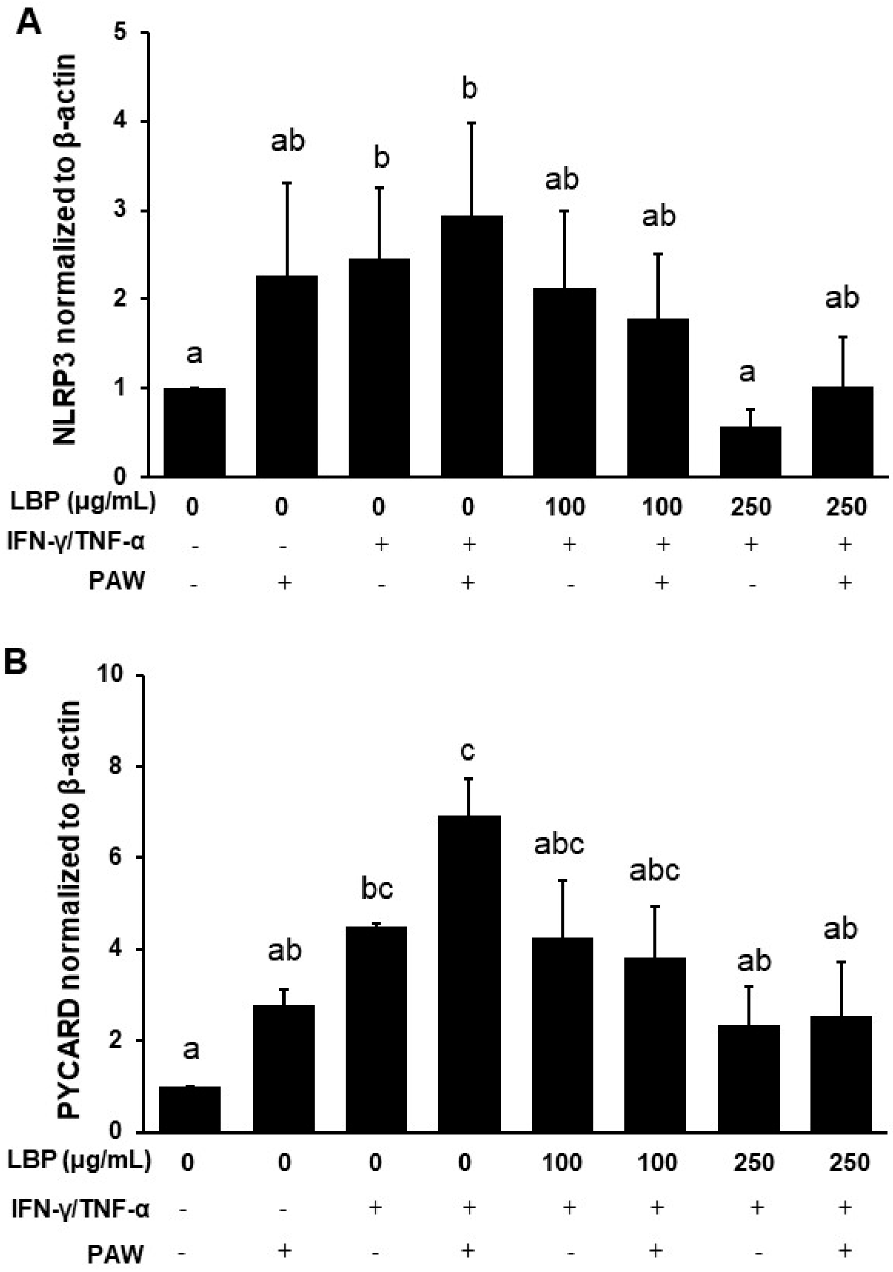

Figure S3. Effects of *Lycium barbarum* polysaccharides (LBP, 100-250 μg/mL) with or without plasmon-activated water (PAW) on protein expression of (**A**) NLRP3 inflammasomes and (**B**) PYCARD in Caco-2 cells. Values are expressed as mean ± SEM (n = 5 per group). The data not sharing the same letter represent significant differences between the groups at $p < 0.05$.

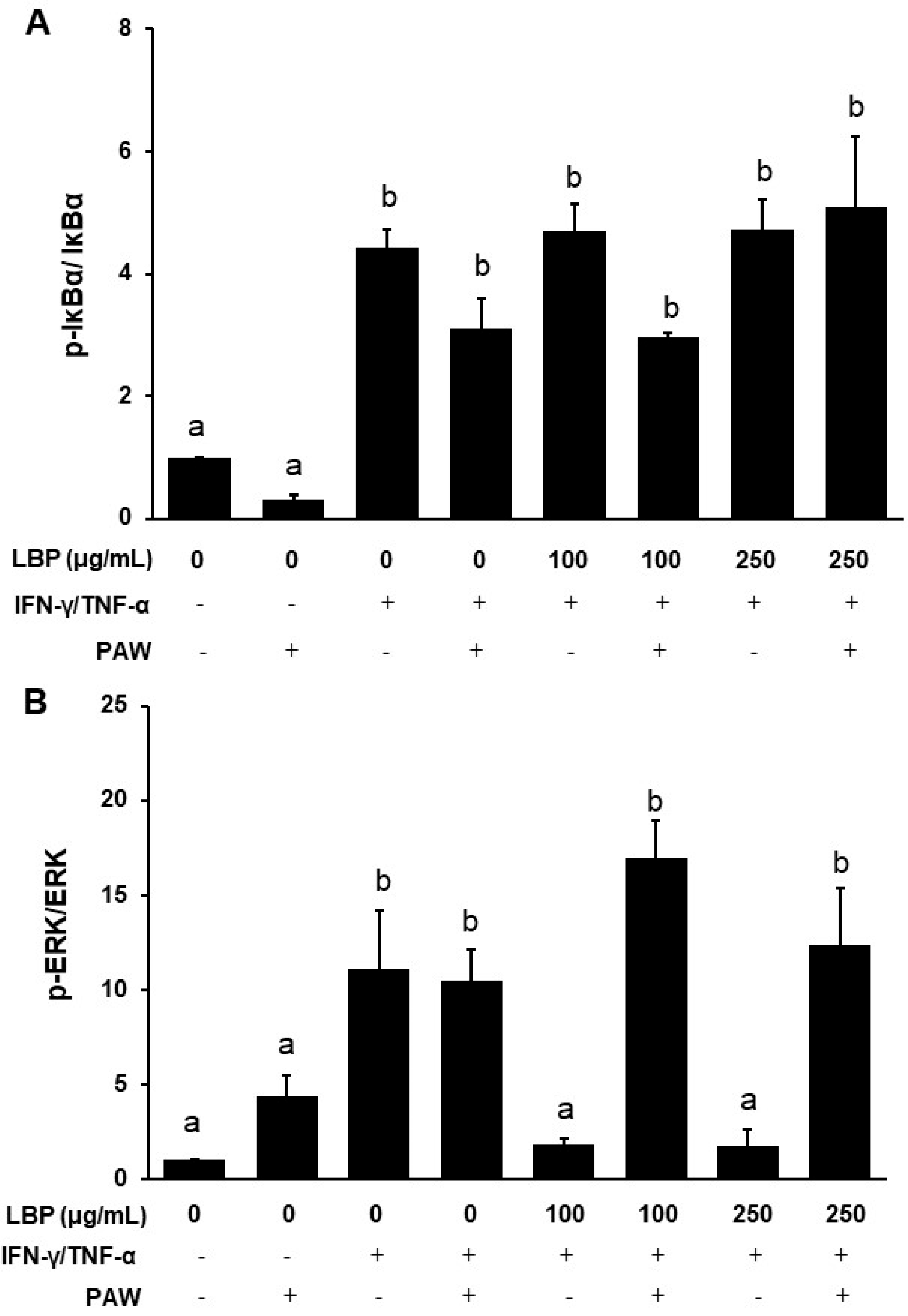

Figure S4. Effects of *Lycium barbarum* polysaccharides (LBP, 100-250 μg/mL) with or without plasmon-activated water (PAW) on (**A**) the ratio of phosphorylated IκBα to total IκBα (p-IκBα/IκBα) and (**B**) the ratio of phosphorylated ERK to total ERK (p-ERK/ERK) protein expression in Caco-2 cells. Values are expressed as mean ± SEM (n = 5 per group). The data not sharing the same letter represent significant differences between the groups at $p < 0.05$.

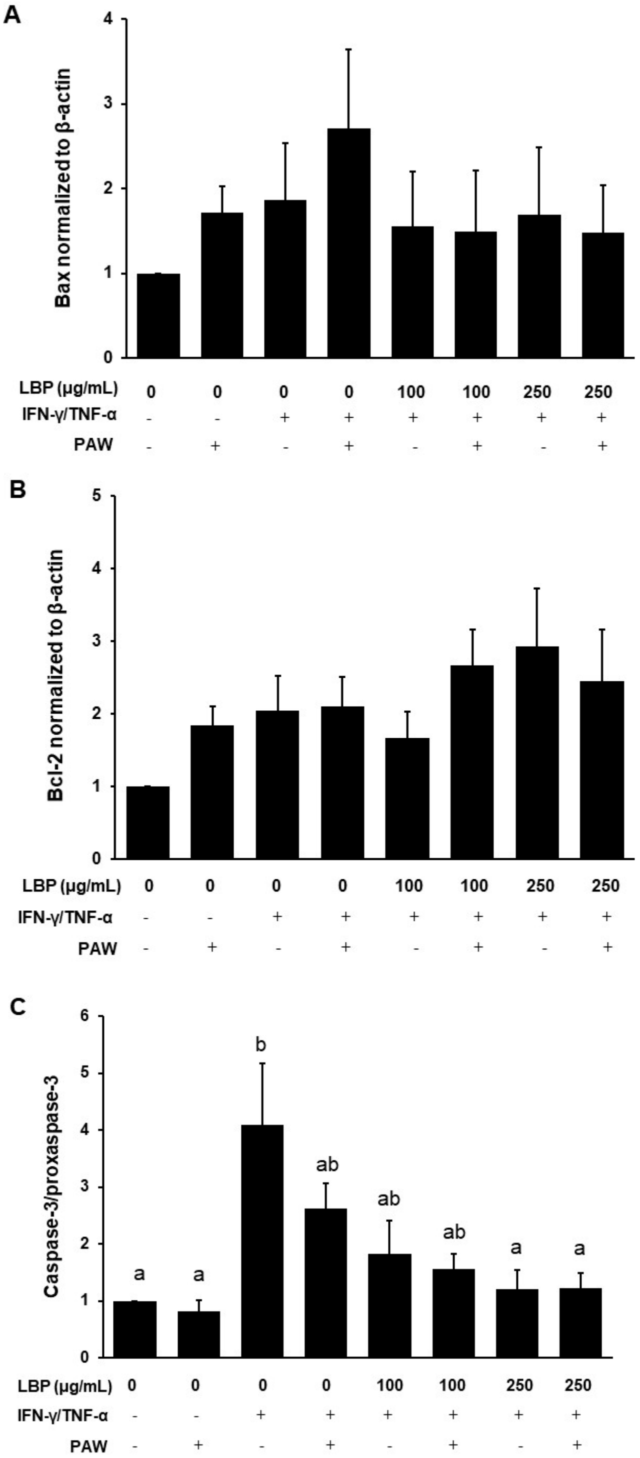

Figure S5. Effects of *Lycium barbarum* polysaccharides (LBP, 100-250 μg/mL) with or without plasmon-activated water (PAW) on protein expression of apoptotic markers (**A**) Bax, (**B**) Bcl-2, and (**C**) caspase-3/procaspase-3 in Caco-2 cells. Values are expressed as mean ± SEM (n = 5 per group). Protein expression of Bax and Bcl-2 was not significantly different among the groups ($p > 0.05$). The data not sharing the same letter represent significant differences between the groups at $p < 0.05$.
